# Optimizing power allocation for URLLC-D2D in 5G networks with Rician fading channel

**DOI:** 10.7717/peerj-cs.2712

**Published:** 2025-02-18

**Authors:** Owais Muhammad, Hong Jiang, Muhammad Bilal, Mushtaq Muhammad Umer

**Affiliations:** 1 School of Information Engineering, Southwest University of Science and Technology, Mianyang, Sichuan, China; 2 School of Software Engineering, Northeastern University, Shenyang, Liaoning, China; 3 Department of Computer Science, University of Pretoria, Pretoria, South Africa

**Keywords:** D2D communication, Rician fading, URLLC, Power allocation

## Abstract

The rapid evolution of wireless technologies within the 5G network brings significant challenges in managing the increased connectivity and traffic of mobile devices. This enhanced connectivity brings challenges for base stations, which must handle increased traffic and efficiently serve a growing number of mobile devices. One of the key solutions to address these challenges is integrating device-to-device (D2D) communication with ultra-reliable and low-latency communication (URLLC). This study examines the impact of the Rician fading channel on the performance of D2D communication under URLLC. It addresses the critical problem of optimizing power allocation to maximize the minimum data rate in D2D communication. A significant challenge arises due to interference issues, as the problem of maximizing the minimum data rate is non-convex, which leads to high computational complexity. This complexity makes it difficult to derive optimal solutions efficiently. To address this challenge, we introduce an algorithm that is based on derivatives to find the optimal power allocation. Comparisons are made with the branch and bound (B&B) algorithm, heuristic algorithm, and particle swarm optimization (PSO) algorithm. Our proposed algorithm improves power allocation performance and also achieves faster execution with lower computational complexity compared to the B&B, PSO, and heuristic algorithms.

## Introduction

The advancements in wireless technology, the number of mobile devices in cellular systems is growing rapidly. As population density in major cities continues to rise, more people are using mobile devices, and the distance between them is shrinking. This has created new possibilities for communication (Banafaa et al., 2023). Technologies such as device-to-device (D2D) communication in 5G have emerged to meet the demand for faster and more efficient information transfer in wireless networks (Papachary, Arya & Dappuri, 2024a). D2D communication eliminates the need for intermediary base stations by enabling direct connection between two devices. It enables devices to create direct connections with one another, facilitating communication without depending on the base station (Borgohain & Choudhury, 2023). D2D communication is an effective solution to address the problem and is being widely embraced in the 5G mobile communications network. Using ultra-reliable and low-latency communication (URLLC) ensures high reliability and low latency for end-to-end communication. Maintaining dependable communication and achieving low latency for optimal control performance is exceedingly challenging. Adopting D2D communication, this approach offers significant advantages, including notable reductions in power consumption, decreased transmission latency, and enhanced overall reliability (Chang et al., 2019; Muhammad et al., 2023). One of the most important components in 5G networks is URLLC and its primary aim is to facilitate the provision of new services and applications that exhibit low latency, availability, and high reliability (Dao et al., 2021). URLLC demands exceptionally stringent criteria, necessitating a 99.999% reliability rate (equivalent to a packet error probability of ${10^{ - 5}}$) (Zheng, Cheng & Liang, 2023) and 1 ms of end-to-end latency (Li et al., 2023).

Conventional wireless networks have been constructed with a major emphasis on long-packet transmission situations in order to optimize power consumption. However, obtaining high reliability and minimal latency in such scenarios is often complex (Adhikari & Hazra, 2022). This emphasizes the importance of creating unique architectures and transmission strategies to meet the URLLC, which show high reliability as well as low latency. D2D communication under URLLC constraints is highly challenging to achieve stringent reliability and low latency (Palla, Amudala & Budhiraja, 2024). In D2D communications, the short proximity between users allows for power allocation, which is not achievable in traditional cellular communications (Salim, Elsayed & Abdalzaher, 2023). D2D communications are increasingly acknowledged as a promising approach to fulfill the rigorous demands of URLLC (Iqbal et al., 2023; Haque et al., 2023). Allocating resources and optimizing power represent significant challenges in D2D communication within cellular networks. D2D communication improves network capacity, optimizes resource allocation, reduces power consumption, and minimizes latency by leveraging user proximity, a capability not achievable in traditional cellular communications (She & Li, 2023; Alibraheemi et al., 2023). The main objective of this research is to explore the impact of Rician fading on uplink D2D communication, focusing on optimizing power allocation to ensure both reliability and low latency under URLLC constraints.

### Contribution

This article focuses on the investigation of Rician fading channels in the propagation of uplink D2D communication in a single-cell cellular environment. System performance in D2D communication can be negatively affected by the influence of a fading environment. Therefore, this study investigates the effect of Rician fading in D2D communication. To address the rigorous QoS demands of D2D communication, the study employs URLLC techniques. The presence of fading in the environment can have an impact on D2D communication, which may lead to a decrease in system performance. It is difficult to solve the minimal rate maximization problem and analysis of the interference. In this study, we find the optimal power allocation for D2D communication by jointly considering reliability and latency constraints in URLLC with low computational complexity. The contributions of this study are outlined as follows:

- An approach has been developed to optimize power allocation for D2D communication within a cellular network. This formulation addresses the challenges of Rician fading and the stringent requirements of URLLC to enhance overall performance.
- The objective is to maximize the minimum rate of D2D users by optimizing power allocation. This problem is non-convex and presents a significant challenge in optimization due to the intricate and non-convex nature of the achievable rate expression.
- An iterative algorithm is proposed, based on derivative, to maximize the minimum rate by efficiently finding the optimal power allocation while maintaining a considerably low computational complexity. The performance of the proposed power allocation algorithm is compared with the branch and bound (B&B) algorithm, particle swarm optimization (PSO) algorithm and heuristic algorithm. The computational complexity of the power allocation algorithm is remarkably lower than that of the B&B, PSO, and heuristic algorithms, while it also outperforms these algorithms in meeting the network’s URLLC requirements.

The subsequent sections of this article are structured as follows. The Related Work section presents a overview of prior research conducted by other scholars in the field. The System Model section presents the system model for the proposed approach, while the Optimizing Power Allocation section formulates the power allocation problem. The simulation results are presented in the Simulation Results and Analysis section, and the last section discusses the conclusions and future work.

## Related work

Recent studies have concentrated on optimizing resource and power allocation in D2D communication within cellular networks. Huang et al. (2024) propose a value decomposition network for resource allocation in D2D communications underlaying cellular networks. The approach reduces interference and improves spectral efficiency by enabling centralized training and distributed decision-making among D2D users. Similarly, Acharjee, Debnath & Arif (2019) proposed an optimal re-source allocation strategy that prioritizes cellular users while enabling resource sharing with D2D pairs. This approach utilized a PSO algorithm to enhance system throughput and reduce power consumption. However, Hussein, Elsayed & Abd El-kader (2020) introduced an approach that considers the Rician fading and minimizes the overall transmit power of the D2D communication, but they did not provide reliable and low-latency communication. Papachary, Arya & Dappuri (2024b) investigate network slicing in D2D networks to improve Enhanced Mobile Broad-band and URLLC. It optimizes energy efficiency and resource allocation through a Mixed-Integer Nonlinear Program that integrates beamforming and resource sharing selection, using methods like the Dinkelbach approach. Jiang et al. (2024) further explore a full-duplex integrated sensing D2D system within cellular networks. They proposes a joint beamforming and power allocation scheme to enhance the performance D2D networks, formulating a non-convex sum rate maximization problem and introduce a successive convex algorithm introduced to solve this problem.

Some studies have examined the D2D communication in URLLC and attempted to get the high quality-of-service (QoS) requirement in URLLC. Elmadina et al. (2023) proposed an interference avoidance algorithm for power allocation in D2D communication. The proposed algorithm minimize interference between D2D users while optimizing power allocation to enhance communication performance. Similarly, Alruwaili, Kim & Oluoch (2024) introduced a method for optimizing power allocation and throughput in 5G cellular systems using D2D communication and a modified Gale-Shapley algorithm. Veedu & Manjappa (2024) proposed frameworks for many-to-many resource allocation and optimal power control in underlay cellular D2D communication to enhance public safety applications. These proposed methods improve the system sum rate and power control. Sanusi & Nasr (2024) introduced a matching theory approach for resource allocation in D2D communication to meet URLLC requirements. Similarly, Kai et al. (2023) introduced a reinforcement learning approach for power allocation in D2D networks. A mode-switching scheme was introduced to optimize power allocation in D2D communications within 5G networks, aiming to maximize joint channel capacity (Gao et al., 2023). Chang et al. (2021) addressed the optimization problem of transmit power in D2D communication for real-time wireless control systems with URLLC and control requirements, a probability-based D2D technique that uses Rayleigh fading, which is less efficient than Rician fading in reducing power consumption.

## System model

**Figure 1:**
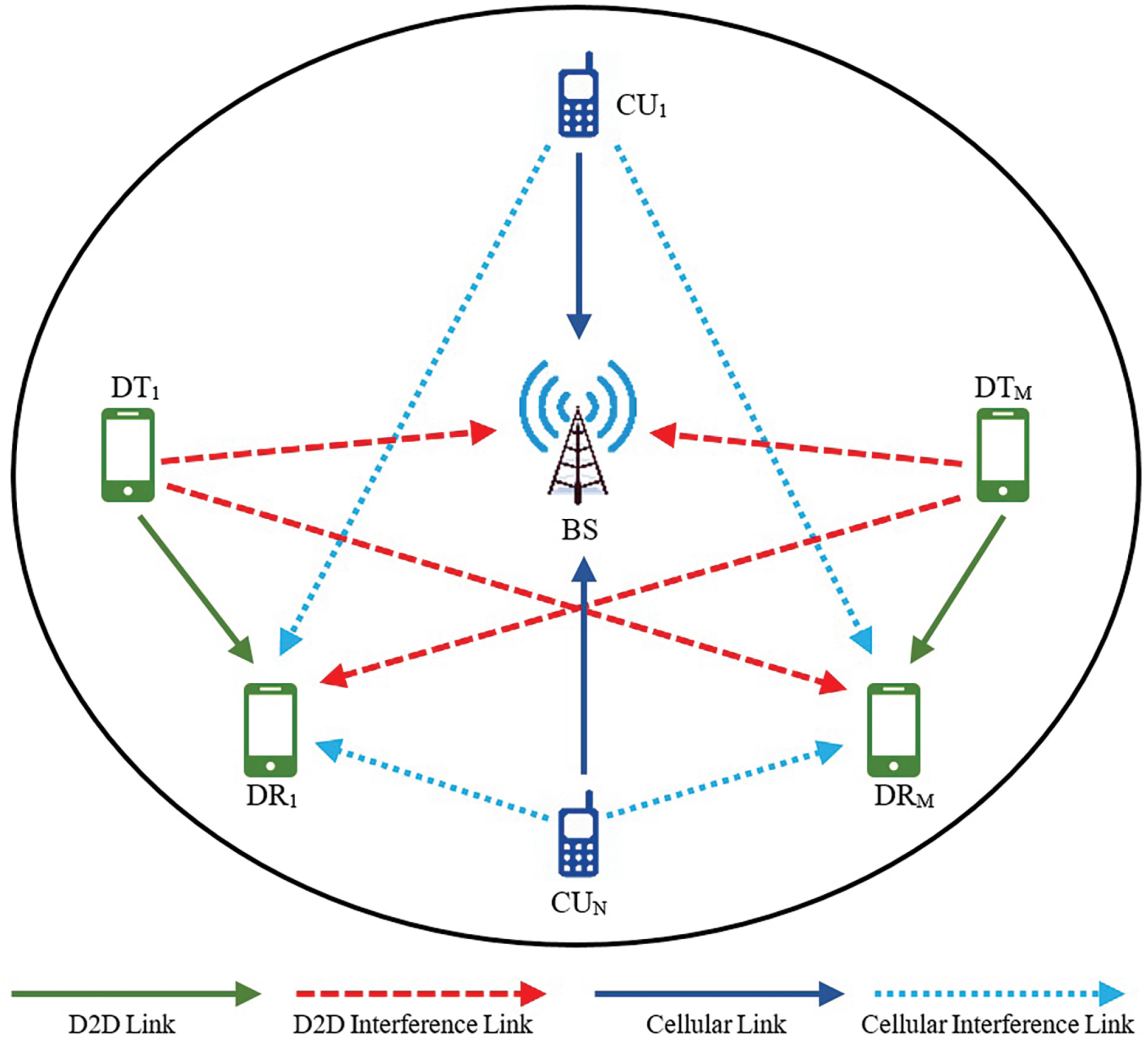
System model.

We investigate uplink D2D communication in a single-cell network of a cellular system, as illustrated in Fig. 1. The cellular base station (BS) is located in the center of the cell, and the cellular users (CUs) are uniformly distributed across the entire cell area. We consider that there are *M* D2D pairs and *N* cellular users.

The sets of cellular users and D2D pairs are denoted by ${\scr L} = \;\left\{ {1,\;2,\; \ldots ,\;N} \right\}$ and ${\scr D} = \;\left\{ {1,\;2,\; \ldots ,\;M} \right\}$ where $M \le N$. The resource reuse indicator, denoted as ${p_{n,m}} \in \left\{ {0,1} \right\}$. Specifically, ${p_{n,m}} = 1$ if D2D pair $m$ reuses the channel of cellular user $n$, otherwise ${p_{n,m}} = 0$ (Xu et al., 2021). The known constants, assuming slow fading components, while the fast-fading components are represented by random variables. $G_m^d$ and $G_{n,m}^{cd}$ are fast-fading coefficients. The interference caused by other D2D connections is denoted as $G_{n,m}^{cd}$ and follows an exponential distribution with a mean of zero and a unit variance. The D2D transmitter (DT) $m$ toward the receiver is expressed as $S_m^d$ is slow fading coefficient, and the power gain of the interference channel between the cellular user $n$ and the D2D receiver (DR) *m* is denoted as $S_{n,m}^{cd}$. Due to strong line-of-sight (LoS) components, we model the communication channel using a Rician fading model, which is suitable for environments where a dominant direct LoS signal exists along with scattered multipath components. The probability density function (PDF) of $G_m^d$ is show in Eq. (1), The Rician *K*-factor quantifies the ratio of the power in the LoS component to the scattered components, with higher values indicating a stronger LoS path and ${I_0}$ is the zeroth-order Bessel function (Peng et al., 2014; Zhang et al., 2024).

(1) $${f_{G_m^d}}\left( z \right) = {{\left( {1 + K} \right){e^{ - K}}} \over {\bar z}}\;exp\left( { - {{\left( {1 + K} \right)z} \over {\bar z}}\;} \right){I_0}\;\left( {2\sqrt K \sqrt {{{\left( {1 + K} \right)z} \over {\bar z}}} } \right).$$

Due to their shared spectrum of resources, D2D users encounter interference from cellular users as well as other D2D pairings. To assess the signal quality, we calculate the SINR, which measures the power of the desired signal relative to interference and noise. A higher SINR value indicates better signal reception (Pan & Zheng, 2023). The Eq. (2) expresses the SINR for the ${m^{th}}$ D2D link. $\;P_n^c$ denotes the transmit power of cellular connection $n$, and the transmit power of D2D link $m$ is represented as $P_m^c$. The additive noise power is denoted as ${\sigma ^2}$.

(2) $$\gamma _m^d = {{P_m^d\;S_m^d\;G_m^d} \over {\mathop \sum \nolimits_{n \in L} {p_{n,m}}P_n^c\;S_{n,m}^{cd}\;G_{n,m}^{cd} + {\sigma ^2}}}.$$

In scenarios involving URLLC, achieving low latency requires users to transmit short packets. The D2D communication achievable rate is given as:

(3) $$R_{m}={\displaystyle{B_m}\over{\rm In \;2}}\left[C_m-\sqrt{\displaystyle{V_m}\over{T_m\; B_m}}fQ^{-1}(\epsilon_m)\right]$$

where ${B_m}$ represents the bandwidth and ${V_m}$ corresponds to the channel dispersion. ${T_m}$ refers to the transmission time delay, and ${C_m}$ represents the Shannon capacity. $f{Q^{ - 1}}( \cdot )$ is the inverse of the *Q* function and $\epsilon_m$ is the transmission error probability. The Shannon capacity is formulated as follows, relying on the received SINR.

(4) $${C_m} = \log \;\left( {1 + \;\gamma _m^d} \right)$$

where ${V_m}$ is the channel dispersion (Chang et al., 2019), it can be expressed as

(5) $${V_m} = \;\left( {1 - {1 \over {{{\left( {1 + \gamma _m^d} \right)}^2}}}} \right) \approx 1$$

When SINR exceeds $5\;dB$ then ${V_m}\; \approx 1$ (Sun, She & Yang, 2023). The analytical expression for the outage probability of D2D communication is given as follows.

(6) $$P_m^{OP} = \Pr \left[ {\gamma _m^d \le {\gamma _0} = {{{\gamma _d}} \over {{\gamma _I}}} \le {\gamma _0}\;or\;{\gamma _d} \le \;{\gamma _{th}}\;} \right].\;$$

The minimal instantaneous signal power threshold is denoted as ${\gamma _{th}}$, whereas the minimal SINR threshold is represented as ${\gamma _0}$. The signal power, represented as ${\gamma _d}$, where ${\gamma _d} = P_m^dS_m^dG_m^d$ and the total interference power, denoted as ${\gamma _I}$, where ${\gamma _I} = \sum\nolimits_{n \in L} {{p_{n,m}}} P_n^c\;S_{n,m}^{cd}G_{n,m}^{cd}$. The symbol ${\gamma _I}$ encompasses both co-tier and cross-tier interference arising from interference among interference from DTs directed toward other DRs and cellular users. ${L_I}$ is the sum of the instantaneous powers, equal to the total instantaneous interference power, which is represented as ${\gamma _I}$, where ${\gamma _I} = \mathop \sum \nolimits_{i = 1}^{{L_I}} {\gamma _i}$ (Huq, Mumtaz & Rodriguez, 2016). The D2D outage probability can be formulated according to Eq. (6) as follows:

(7) $$P_m^{OP} = \left\{ {\gamma _m^d \le {\gamma _0}} \right\} = \Pr \left( {{{P_m^d\;S_m^d\;G_m^d} \over {\mathop \sum \nolimits_{n \in L} {p_{n,m}}P_n^c\;S_{n,m}^{cd}G_{n,m}^{cd}}} \le \;{\gamma _0}} \right).$$

The power of interference from the cellular user to the D2D receiver is generally much greater than the power of noise (Peng et al., 2014). We assume that D2D links are interference-limited, and we can ignore the influence of noise power on the outage probability (Yin et al., 2015). Equation (7) is represented in the form of a PDF as follows:

(8) $$P_m^{OP} = 1 - \int_{{\gamma _{th}}}^\infty \left( {\mathop \int \limits_0^{{{{\gamma _d}} \over {{\gamma _0}}}} {f_{{\gamma _I}}}\left( {{\gamma _I}} \right)d{\gamma _I}} \right){f_{{\gamma _d}}}\left( {{\gamma _d}} \right)d{\gamma _d}.$$

The PDF of the instantaneous signal power is represented as ${f_{{\gamma _d}}}\left( {{\gamma _d}} \right)$, and the total interference power PDF is represented as ${f_{{\gamma _I}}}\left( {{\gamma _I}} \right)$. The received signal from the intended user conforms to a Rician distribution, and there is Rayleigh interference in the system that is ${L_I}$ i.i.d. The total instantaneous interference power ${\gamma _I}$ PDF can be denoted as

(9) $${f_{{\gamma _I}}}\left( {{\gamma _I}} \right) = {{{\gamma _I}^{{L_I} - 1}} \over {\bar \gamma _I^{{L_I}}\left( {{L_I} - 1} \right)!}}exp\left( { - {{{\gamma _I}} \over {{{\bar \gamma }_I}}}} \right).$$

The statistical average of ${\gamma _I}$ is denoted as ${\bar \gamma _I}$, and the instantaneous signal power ${\gamma _d}$ PDF denoted as

(10) $${f_{{\gamma _d}}}\left( {{\gamma _d}} \right) = {{\left( {K + 1} \right)} \over {{{\bar \gamma }_d}}}\;{e^{\left[ { - K - {{\left( {K + 1} \right){\gamma _d}} \over {{{\bar \gamma }_d}}}} \right]}}{I_0}\;\left( {2\sqrt {{{K\left( {K + 1} \right){\gamma _d}} \over {{{\bar \gamma }_d}}}} } \right).\;$$

By applying Eqs. (9) and (10), the outage probability of D2D can be reformulated within Eq. (8)

(11) $$\eqalign{& {P_m^{OP} = 1 - \int_{{\gamma _{th}}}^\infty  \left( {\mathop \int \limits_0^{{{{\gamma _d}} \over {{\gamma _0}}}} {{{\gamma _I}^{{L_I} - 1}} \over {\bar \gamma _I^{{L_I}}\left( {{L_I} - 1} \right)!}}exp\left( { - {{{\gamma _I}} \over {{{\bar \gamma }_I}}}} \right)d{\gamma _I}} \right)} \hfill \cr & {\;\left( {{{\left( {K + 1} \right)} \over {{{\bar \gamma }_d}}}\;{e^{\left[ { - K - {{\left( {K + 1} \right){\gamma _d}} \over {{{\bar \gamma }_d}}}} \right]}}{I_0}\left( {2\sqrt {{{K\left( {K + 1} \right){\gamma _d}} \over {{{\bar \gamma }_d}}}} } \right)} \right).} \hfill \cr  }$$

We obtain the outage probability, by solving the inner integral of Eq. (11). This indicates that the outage probability is influenced by the Rician *K*-factor, the signal power, and the total interference power ${\gamma _I}$. Increasing the strength of the LoS component (higher *K*-factor) or reducing the total interference can lower the outage probability, thereby enhancing communication reliability.

(12) $$P_m^{OP} = {{exp\left[ { - K + {K \over {\left( {1 + {{{{\bar \gamma }_d}} \over {\left( {K + 1} \right){{\bar \gamma }_d}}}} \right)}}} \right]} \over {\left( {1 + {{{{\bar \gamma }_d}} \over {\left( {K + 1} \right){\gamma _0}{{\bar \gamma }_I}}}} \right)}}.$$

Proof: See Appendix A.

The ergodic capacity of ${C_m}$, derived from the overall performance across all channel fading states, can be expressed as follows

(13) $${C_m} = \;E\;\left[ {\log \;\left( {1 + \;\gamma _m^d\;} \right)} \right]\;$$

(14) $$E\;\left[ {\log \;\left( {1 + \;\gamma _m^d\;} \right)} \right]\; = \;\mathop \int \limits_0^\infty log\left( {1 + \;x} \right)\;{f_{\gamma _m^d}}\left( x \right)dx.$$

Over the fast-fading distribution, the expectation $E[\cdot]$ is taken. By applying integration-by-parts

(15) $$\mathop \int \limits_{x = 0}^\infty \mathop \int \limits_{y = 0}^x {1 \over {1 + y}}{f_{\gamma _m^d}}\left( x \right)dydx$$

(16) $$\mathop \int \limits_{y = 0}^\infty {1 \over {1 + y}}dy\mathop \int \limits_{x = y}^\infty {f_{\gamma _m^d}}\left( x \right)dx.$$

The ergodic capacity is calculated by the following expression:

(17) $$C_m^* = \mathop \int \limits_0^\infty {{1 - {{\mathrm{F}}_{\gamma _m^d}}\left( x \right)} \over {1 + x}}\;dx.\;$$

where ${{\mathrm{F}}_{\gamma _m^d}}\left( x \right) = \Pr \left( {\gamma _m^d \le {\gamma _0}} \right)$ is provided in Eq. (12), then Eq. (3) can be reformulated as:

(18) $$R_m^* = \;{{{B_m}} \over {ln2}}\;\left[ {C_m^* - \sqrt {{{{V_m}} \over {{T_m}{B_m}}}} \;f{Q^{ - 1}}\left( {{\epsilon _m}} \right)} \right].$$

Equation (19) provides an expression for the probability of a packet error, ${h_m} = \;R_m^*{T_m}$ represents the amount of bits that must be transferred during each transmission.

(19) $$\epsilon _m^* = fQ\left\{ {\sqrt {{{{T_m}{B_m}\;} \over {{V_m}}}} \;\left[ {C_m^* - \;{{{h_m}ln2} \over {{T_m}{B_m}}}} \right]} \right\}.$$

The following constraints must be satisfied in order to satisfy URLLC reliability requirements.

(20) $$\epsilon _m^* \le \;{\epsilon _{max}}.$$

The maximum packet error probability denoted as ${\epsilon _{max}}$, is restricted by the Quality of Service requirements for URLLC and additionally imposes a constraint on the time delay for communication such as

(21) $${T_m} \le {T_{max}}.$$

The successful transmission probability can be formulated by considering the reliability constraint in URLLC such as

(22) $$1 - \epsilon _m^* \ge 1 - {\epsilon _{max}}.$$

The transmit power that meets the constraints can be derived from Eq. (3) as follows

(23) $${R_m} = \;{{{B_m}} \over {ln2}}\;\left[ {{C_m} - \sqrt {{1 \over {{T_m}{B_m}}}} \;f{Q^{ - 1}}\left( {\epsilon _m^*} \right)} \right]$$

(24) $${{{R_m}{T_m}ln2} \over {{T_m}{B_m}}} = {C_m} - \sqrt {{{{V_m}} \over {{T_m}{B_m}}}} \;f{Q^{ - 1}}\left( {\epsilon _m^*} \right)$$

(25) $${C_m} = {{{h_m}ln2} \over {{T_m}{B_m}}} + \sqrt {{{{V_m}} \over {{T_m}{B_m}}}} \;f{Q^{ - 1}}\left( {\epsilon _m^*} \right)$$

where ${C_m} = \;\log \;\left( {1 + \;\gamma _m^d\;} \right)$

(26) $$\log \left( {1 + \;{{P_m^dS_m^d\;G_m^d} \over {\mathop \sum \nolimits_{n \in {\scr L}} {p_{n,m}}P_n^cS_{n,m}^{cd}G_{n,m}^{cd} + {\sigma ^2}}}\;} \right) = {{{h_m}ln2} \over {{T_m}{B_m}}} + \sqrt {{1 \over {{T_m}{B_m}}}} f{Q^{ - 1}}\left( {\epsilon _m^*} \right)$$

(27) $$1 + {{P_m^dS_m^d\;G_m^d} \over {\mathop \sum \nolimits_{n \in {\scr L}} {p_{n,m}}P_n^cS_{n,m}^{cd}G_{n,m}^{cd} + {\sigma ^2}}} = exp\;\left[ {{{{h_m}ln2} \over {{T_m}{B_m}}} + \;\sqrt {{1 \over {{T_m}{B_m}}}} f{Q^{ - 1}}\left( {\epsilon _m^*} \right)} \right]$$

(28) $$P_m^d = {{\mathop \sum \nolimits_{n \in {\scr L}} {p_{n,m}}P_n^cS_{n,m}^{cd}G_{n,m}^{cd} + {\sigma ^2}} \over {S_m^d\;G_m^d}}\left\{ {exp\;\left[ {{{{h_m}ln2} \over {{T_m}{B_m}}} + \;\sqrt {{1 \over {{T_m}{B_m}}}} f{Q^{ - 1}}\left( {\epsilon _m^*} \right)} \right] - 1} \right\}$$

where ${{\mathop \sum \nolimits_{n \in {\scr L}} {p_{n,m}}P_n^cS_{n,m}^{cd}G_{n,m}^{cd} + {\sigma ^2}} \over {S_m^d{\mathrm{ }}\;{\mathrm{ }}G_m^d}}$ solved by outage probability Eq. (7)

(29) $$P_m^d = P_m^{OP}\left\{ {exp\;\left[ {{{{h_m}ln2} \over {{T_m}{B_m}}} + \;\sqrt {{1 \over {{T_m}{B_m}}}} f{Q^{ - 1}}\left( {\epsilon _m^*} \right)} \right] - 1} \right\}.$$

Equation (29) represents the transmit power $P_m^d$ required to meet certain constraints while considering the outage probability $P_m^{OP}$ in D2D communication. It shows the relationship between transmit power, the required signal quality, and the interference experienced in the system.

## Optimizing power allocation

In this section, we present the optimization problem with the objective of maximizing the minimum achievable rate for D2D users, with a specific emphasis on optimizing power allocation. The formulation is presented as follows:

$${\mathrm{P}}1:\matrix{ {{\mathrm{max}}\;\;\;{\mathrm{min}}\;\;} \cr  {P_m^d\;m\;} \cr  } \left\{ {R_m^*} \right\}$$

(30a) $$s.t\quad \epsilon _m^* \le {\epsilon _{max}},\quad \forall m \in {\scr D}$$

(30b) $${T_m} \le {T_{max}},\quad \forall m \in {\scr D}$$

(30c) $$Pr\left\{ {\gamma _m^d \le {\gamma _0}} \right\} \le {\delta _0},\quad \forall m \in {\scr D}$$

(30d) $$\sum\nolimits_{m \in {\scr D}} {P_m^d} \le P_{max}^d.$$

This optimization problem is to find the total optimal transmit power $\sum\nolimits_{m \in {\scr D}} P_m^d$, in order to maximize the minimum achievable rate. Equation (30a) is utilized to ensure the D2D users’ reliability. The utilization of the constraint expressed in Eq. (30b) aims to enforce a limitation on the transmission time delay, ensuring that it does not exceed the maximum transmission time delay ${T_{max}}$. In Eq. (30c), ${\delta _0}$ represents the maximum allowable outage probability constraint and, Eq. (30d) defines the transmit power constraint, which imposes limitations on the total transmit power. $P_{max\;}^d$ denotes the maximum transmit power.

We minimize the total power consumption $\sum\nolimits_{m \in {\scr D}} \; P_m^d$. The following problem can be solved to obtain the minimum total transmit power.

$${\mathrm{P}}2:\matrix{ {{\mathrm{max}}\;\;\;{\mathrm{min}}\;\;} \cr  {P_m^d\;m\;} \cr  } \left\{ {R_m^*} \right\}$$

(31a) $$s.t\quad \epsilon _m^* \le {\epsilon _{max}},\quad \forall m \in {\scr D}$$

(31b) $${T_m} \le {T_{max}},\quad \forall m \in {\scr D}$$

(31c) $$P_m^d \ge 0,\quad \forall m \in {\scr D}$$

(31d) $$Pr\left\{ {\gamma _m^d \le {\gamma _0}} \right\} \le {\delta _0},\quad \forall m \in {\scr D}$$

(31e) $$\sum\nolimits_{m \in {\scr D}} P_m^d \le P_{max}^d.$$

Equation (31c) guarantees that each user is allocated a non-negative power. Due to interference, the achievable rate is non-convex, as shown in Eqs. (2) and (18), which makes the power allocation difficult to solve. We use an algorithm based on derivative to address the non-convex function in order to overcome this challenge. The best possible solution can be reached through the resolution of the provided set of equations.

(32) $${R_m}\;{\triangleq}{f_m}\left( {P_m^{d*}} \right) = {{{B_m}} \over {ln2}}\;\left[ {{C_m} - \sqrt {{1 \over {{T_m}{B_m}}}} \;f{Q^{ - 1}}\left( {\epsilon _m^*} \right)} \right]$$

(33) $${{d{f_m}\left( {P_m^{d*}} \right)} \over {dP_m^{d*}}} = {{{B_m}} \over {ln2}}\left( {{{P_m^{OP*}} \over {1 + \;P_m^{OP}}}} \right)$$

Equation (33) represents the derivative of the function ${f_m}\left( {P_m^{d*}} \right)$ with respect to the transmit power $P_m^{d*}$, is provided in Appendix B.

### Proposed power allocation optimization algorithm

**Figure 2:**
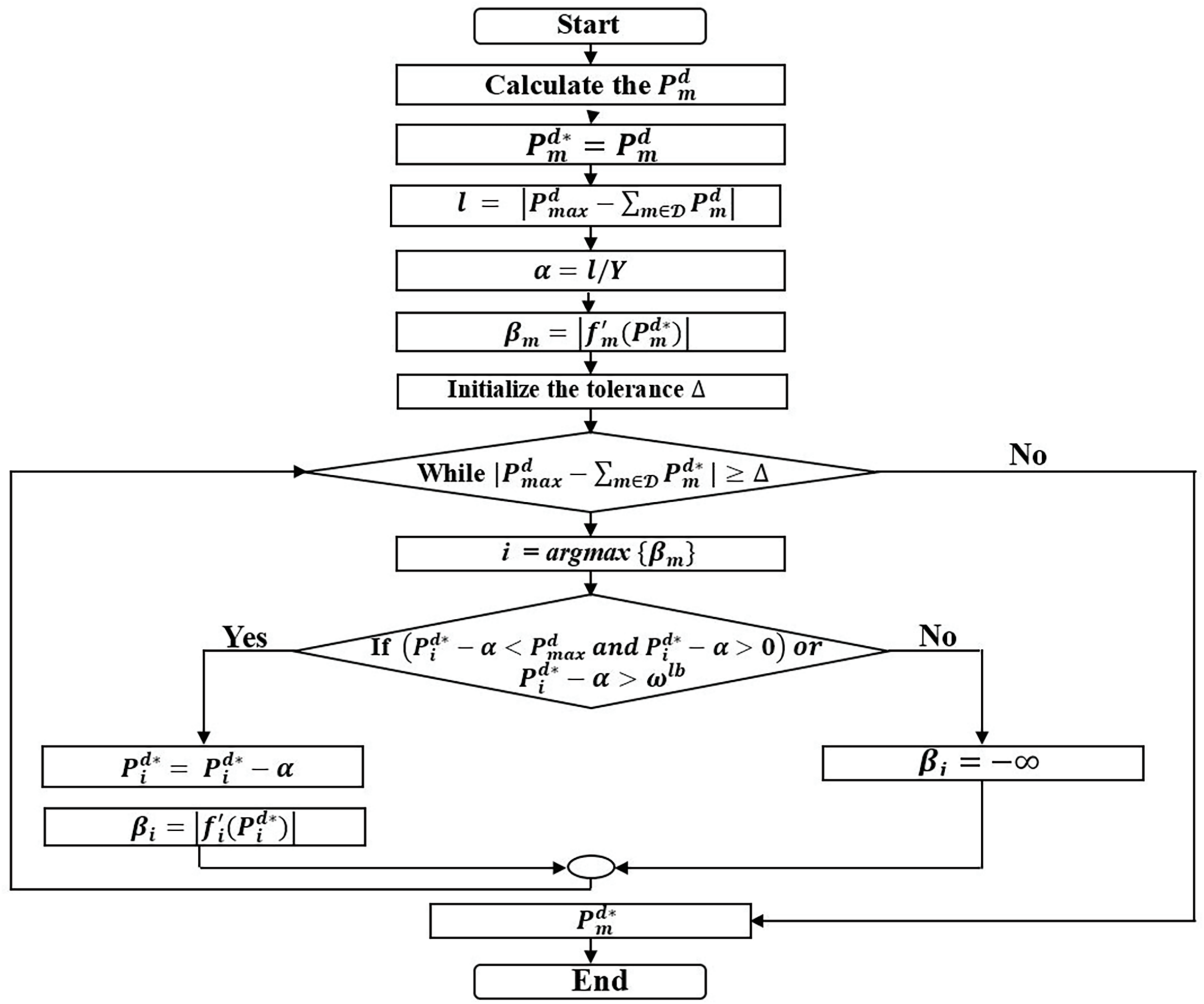
Flowchart of the proposed power allocation optimization algorithm.

The proposed power allocation algorithm, based on derivative is used to identify the optimal solution for the power allocation problem in D2D communication while maintaining the QoS requirements of URLLC. The algorithm is designed to ensure low computational complexity. The flowchart in Fig. 2 presents an iterative algorithm for optimizing power allocation. The algorithm begins by calculating the initial power allocation for each D2D device using Eq. (29) and sets this as a starting point. The algorithm then iteratively adjusts the power allocation based on the difference between total allocated power and the maximum limit, using a step size $\alpha$ controls the adjustment to the power values in each iteration and derivative ${\beta _m}$ to guide the adjustments. The loop continues until the total power is within a tolerance $\Delta$, ensuring an optimal solution with low computational complexity. The following key steps are used to describe the power allocation algorithm:

In steps 1–3, Calculate the $P_m^d$ by using Eq. (29) and set to $P_m^{d*}$. Subtract the maximum power, $P_{max}^d$ from the sum of the calculated power, $\sum\nolimits_{m \in {\scr D}} P_m^d$ and assign it to $l$ as follows $l = \left| {P_{max}^d - \sum\nolimits_{m \in {\scr D}} P_m^d} \right|$.

In step 4, $\alpha = {l \over Y}$, where $\alpha$ is the adjustment step of $P_m^{d*}$ and *Y* is the controlling factor for $\alpha$ and also controls the performance and computational complexity. $l$ represents the difference between the maximum allowable power $P_{max}^d$ and the sum of the current power values. Allowing us to assign an appropriate value based on the specific practical requirements and the lower bound feasible region is set to ${\omega ^{lb}} = 0$.

In step 5, we initialize a variable ${\beta _m}$, to store the derivative of $\left| {f_m^\prime \left( {P_m^{d*}} \right)} \right|$. This variable is utilized in step 8 to select the suitable $P_m^{d*}$. It may also undergo updates in step 11 or step 13 during each iteration of the algorithm.

In steps 6, to decide when to end the loop, we set a tolerance threshold $\Delta$ rather than use a counter, which determines when to stop the algorithm. The loop will continue adjusting the power allocation until the difference between $P_{max}^d$ and the total allocated power is less than $\Delta$.

In steps 7–8, if during the current iteration, the selected $P_i^{d*}$ exceeds the feasible region even after adjustment, then it needs to select another $P_m^{d*}$ for adjustment after this iteration. The loop continues as long as the total power allocation difference $P_{max}^d - \sum\nolimits_{m \in {\scr D}} P_m^{d*}$ is greater than $\Delta$.

In steps 9–11, provided the feasible region to optimize the $P_i^{d*}$ and instead of updating every ${\beta _m}$ for $m \in {\scr D}$ and $m \ne i$, we update only ${\beta _i}$.

In step 13, if the value of $P_i^{d*}$ exceeds the feasible region after adjustment, we assign an infinite to ${\beta _i}$ to ensure that $i$ will not be selected again in step 8, this can prevent the possibility of an infinite loop. The power allocation algorithm solves the problem P2, as shown in the power allocation algorithm.

**Algorithm 1:** Power allocation optimization algorithm.

**Input:** $\Delta ,P_{max}^d,\;{\omega ^{lb}}$
**Output:** $P_m^{d*}$
1: Calculate the $P_m^d$ by using Eq. (29);
2: $P_m^{d*} = P_m^d;$
3: $l = \left| {P_{max}^d - \sum\nolimits_{m \in {\scr D}} P_m^d} \right|$;
4: $\alpha = {l \over Y}$;
5: ${\beta _m} = \left| {f_m^\prime \left( {P_m^{d*}} \right)} \right|$;
6: Initialize the tolerance threshold Δ to control the loop;
7: **while** $\left| {P_{max}^d - \sum\nolimits_{m \in {\scr D}} P_m^{d*}} \right| \ge \Delta$ **do**
8: $i = \arg {\mathrm{max}}\left\{ {{\beta _m}} \right\}$;
9: **if** $\left( {P_i^{d*} - \alpha \; < \; P_{max}^d\;and\;P_i^{d*} - \alpha \; > \; 0} \right)\;{\bf{or}}\;P_i^{d*} - \alpha \; > \; {\omega ^{lb}}$ **then**
10: $P_i^{d*} = \;P_i^{d*} - \alpha$;
11: ${\beta _i} = \left| {f_i^\prime \left( {P_i^{d*}} \right)} \right|$;
12: **else**
13: ${\beta _i} = - \infty$;
14: **end if**
15: **end while**

### Complexity analysis

In the proposed power allocation algorithm, computational complexity is determined by the adjustments made in step 9 and step 13 from the power allocation algorithm, if in the current iteration, the selected ${\mathrm{ }}\;{\mathrm{ }}P_i^{d{\mathrm{*}}}$ exceeds the feasible region after adjustment, ${\beta _i}{\mathrm{ }}\;{\mathrm{ }}$ will be assigned to an infinite. This ensures that the same ${\mathrm{ }}\;{\mathrm{ }}P_i^{d{\mathrm{*}}}$ will not be selected again, as we always choose the *i* value with the maximum ${\beta _i}{\mathrm{ }}\;{\mathrm{ }}$ in step 8. Consequently, for a specific $i$, the occurrence of the currently chosen ${\mathrm{ }}\;{\mathrm{ }}P_i^{d{\mathrm{*}}}$ being unable to be adjusted due to the constraints of the feasible region will occur at most, only once. Therefore, in the ideal scenario where all the chosen ${\mathrm{ }}\;{\mathrm{ }}P_i^{d{\mathrm{*}}}$ can be adjusted during the iterations, the constraint in P2 will be fulfilled after adjusting ${\mathrm{ }}\;{\mathrm{ }}P_i^{d{\mathrm{*}}}$ for*Y* times. Therefore, the power allocation algorithm computational complexity is $O(Y)$.

## Simulation results and analysis

In this section, we examine and analyze the performance of the proposed power allocation algorithm, conducting a comparative evaluation against the B&B, PSO and heuristic algorithms. The objective of the optimization problem is to maximize the minimum rate through efficient power allocation while considering Rician fading conditions. A key parameter in our analysis is the URLLC QoS, which ensures that stringent requirements for reliability and latency are met, along with the *K*-factor, representing the ratio of direct LoS signal power to multipath components. Higher *K*-factors, with stronger LoS, improve reliability and data rates, while lower *K*-factors increase fading and reduce rates. The power allocation algorithm focuses on minimizing power consumption while meeting the requirements of URLLC. By selecting a range of *K*-factors, from high to low, we evaluate the power allocation algorithm effectiveness in optimizing power allocation in both ideal and challenging channel conditions. The simulation parameters we used are summarized in Table 1.

**Table 1:** Simulation parameters.

Parameters	Values
*K*	$1,2,4,6,8,10$
Cell radius	$500\;m$
D2D distance (min, max)	$\left[ {15\;m,\;50\;m} \right]$
${{\scr L}_N}$	$10$
${{\scr D}_M}$	$8$
Noise spectral density	$- 174\;dBm/Hz$
$P_{max}^d$	$10\;dBm$
${B_{max}}$	$20\;MHz$
Shadowing standard deviation	$10\;dB$
${T_{max}}$	$0.1\;ms$
$\Delta$	$1 \times {10^{ - 3}}$
Cellular D2D links path loss model	$148 + 40log\left( d \right)$
$\epsilon_{\rm max}$	$1 \times {10^{ - 5}}$
${\delta _0}$	$1 \times {10^{ - 2}}$

**Figure 3:**
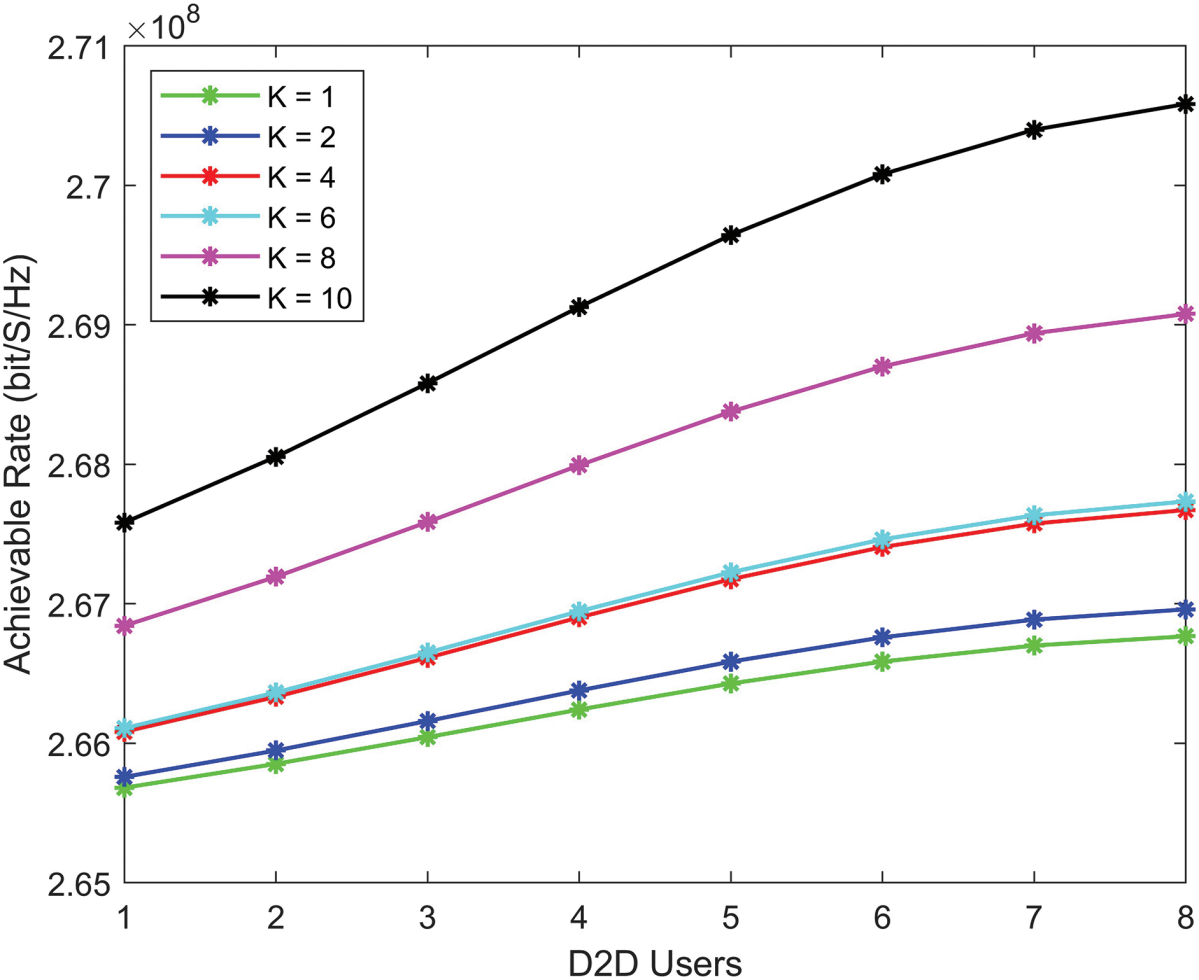
D2D communication achievable rate with various *K* factors (higher *K*-factors providing better performance as the number of users increases).

Figure 3 shows that the achievable rate for D2D communication improves as the Rician *K*-factor increase. This improvement is due to the robust LoS connection and reduced multipath propagation loss. When the *K*-factor is high, D2D users achieve higher data rates as interference is minimized.

**Figure 4:**
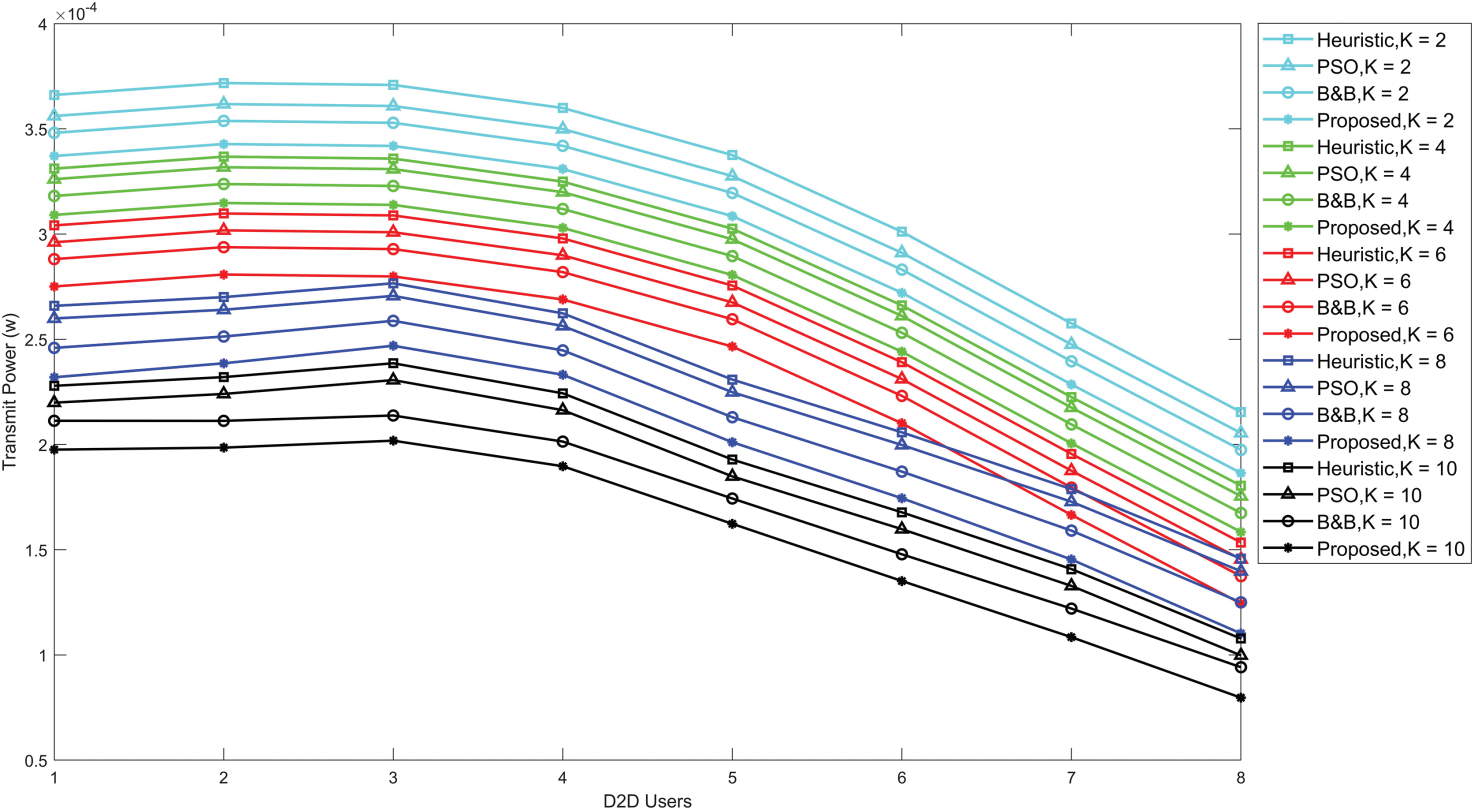
Optimal D2D transmission power under URLLC with various Rician *K*-factors, comparing PSO, B&B, heuristic and the proposed power allocation algorithm.

Figure 4 compares the proposed power allocation algorithm transmit power performance with the B&B, PSO and heuristic algorithms. The evaluation was conducted using various Rician *K*-factors. The results show that the power allocation algorithm delivers the best solution while consuming the least amount of power in D2D communication. Simultaneously, a notable performance gap is observed between the proposed power allocation algorithm, the PSO, the heuristic algorithm, and the B&B algorithm. The optimization of transmit power takes place as the Rician *K*-factor increases. This is due to a higher *K*-factor indicates a stronger LoS component and reduced signal propagation loss resulting from multiple signal paths.

The average computation time (ACT) and average transmit power (ATP) for the proposed power allocation algorithm, the B&B, PSO algorithm and heuristic algorithm with various Rician *K*-factors are shown in Table 2. The power allocation algorithm attains optimal outcomes while maintaining lower computational complexity. The comparative analysis indicates that the proposed power allocation algorithm outperforms the B&B algorithm, PSO algorithm, and heuristic algorithm in terms of speed while also achieving optimal power allocation. Moreover, the heuristic and PSO algorithms, despite their low computational complexity, exhibits faster performance compared to the B&B algorithm.

**Table 2:** ATP (w) and ACT (s) for the proposed power allocation algorithm, B&B algorithm, PSO algorithm and heuristic algorithm.

	Proposed algorithm		B&B algorithm		PSO algorithm		Heuristic algorithm	
*K*	ATP	ACT	ATP	ACT	ATP	ACT	ATP	ACT
1	$3.14 \times {10^{ - 4}}$	5.73	$3.31 \times {10^{ - 4}}$	11.31	$3.41 \times {10^{ - 4}}$	9.15	$3.61 \times {10^{ - 4}}$	7.38
2	$2.91 \times {10^{ - 4}}$	5.63	$3.05 \times {10^{ - 4}}$	11.19	$3.13 \times {10^{ - 4}}$	9.37	$3.23 \times {10^{ - 4}}$	7.33
4	$2.66 \times {10^{ - 4}}$	5.71	$2.75 \times {10^{ - 4}}$	11.39	$3.83 \times {10^{ - 4}}$	9.12	$2.88 \times {10^{ - 4}}$	7.35
6	$2.32 \times {10^{ - 4}}$	5.66	$2.45 \times {10^{ - 4}}$	11.64	$2.53 \times {10^{ - 4}}$	9.13	$2.61 \times {10^{ - 4}}$	7.32
8	$1.98 \times {10^{ - 4}}$	5.65	$2.11 \times {10^{ - 4}}$	11.58	$2.24 \times {10^{ - 4}}$	9.29	$3.30 \times {10^{ - 4}}$	7.28
10	$1.59 \times {10^{ - 4}}$	5.62	$1.72 \times {10^{ - 4}}$	11.13	$1.84 \times {10^{ - 4}}$	9.17	$1.92 \times {10^{ - 4}}$	7.17

**Figure 5:**
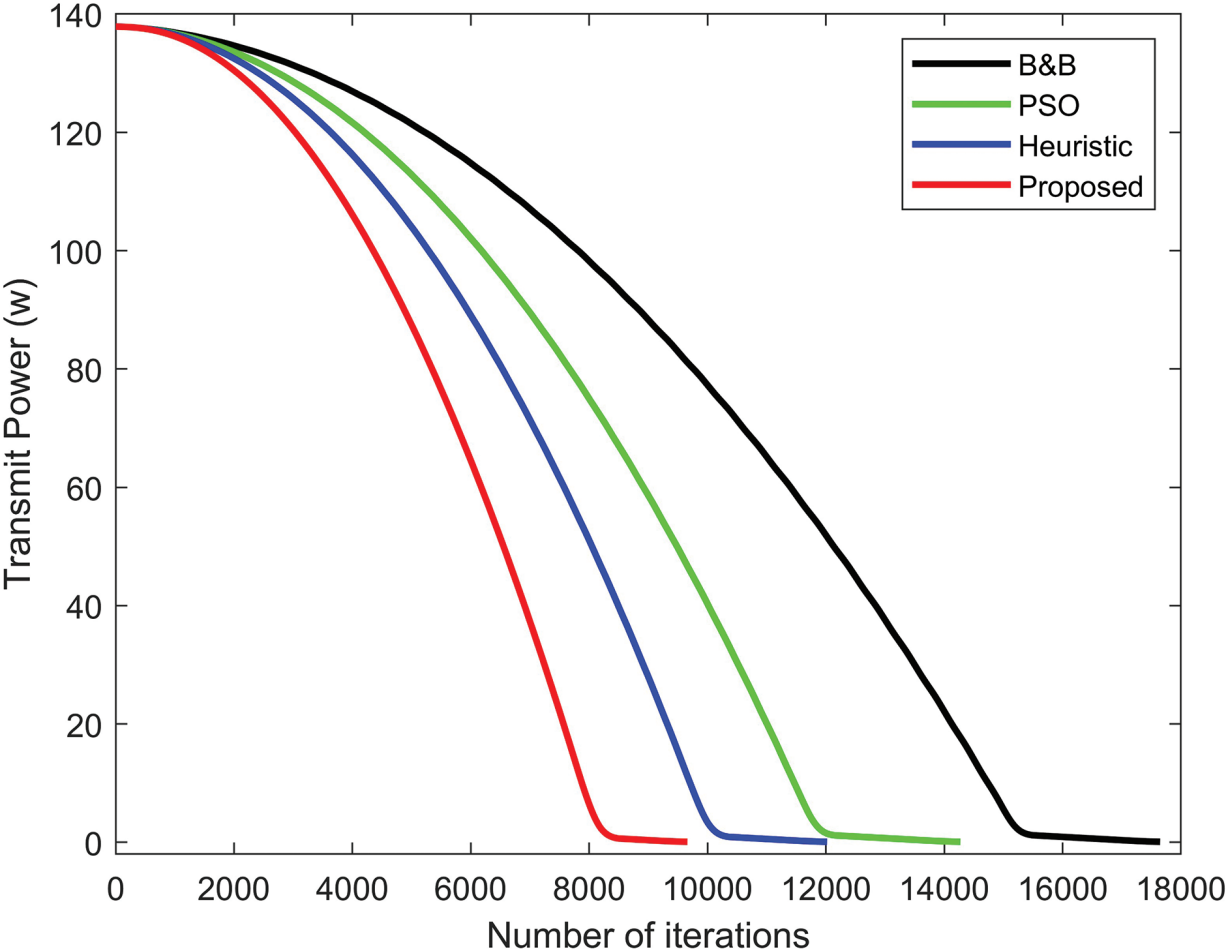
Comparison of transmit power convergence among the B&B algorithm, the PSO algorithm, the heuristic algorithm, and the proposed power allocation algorithm.

**Figure 6:**
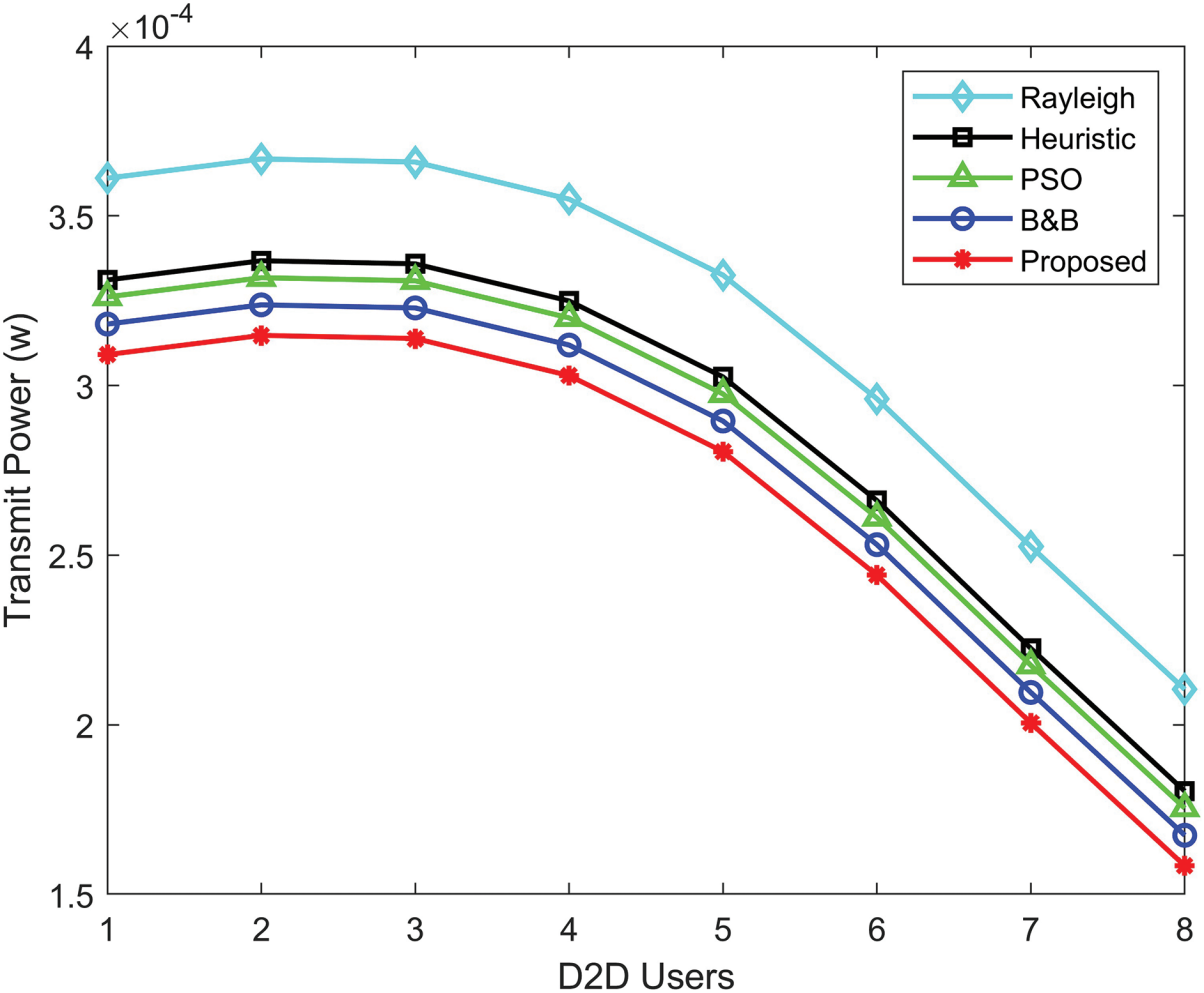
Transmit power comparison for D2D users using Rayleigh, heuristic, PSO, B&B, and proposed power allocation algorithms.

Figure 5 illustrates the convergence behavior of the proposed power allocation algorithm, the PSO algorithm, the heuristic algorithm, and the B&B algorithm. The minimal transmit power for D2D communication achieved by the power allocation algorithm exhibits a rapid decrease in the number of iterations, in comparison to the B&B algorithm and other algorithms. The gap of the power allocation algorithm decreases much more rapidly compared to the other algorithm. Figure 6 compares the transmit power performance of Rayleigh fading with the proposed power allocation algorithm, B&B, PSO, and heuristic algorithms. The proposed algorithm achieves the best performance, consuming the least power in D2D communication due to the advantages of Rician fading. Rayleigh fading performs worse due to the non-line-of-sight.

**Figure 7:**
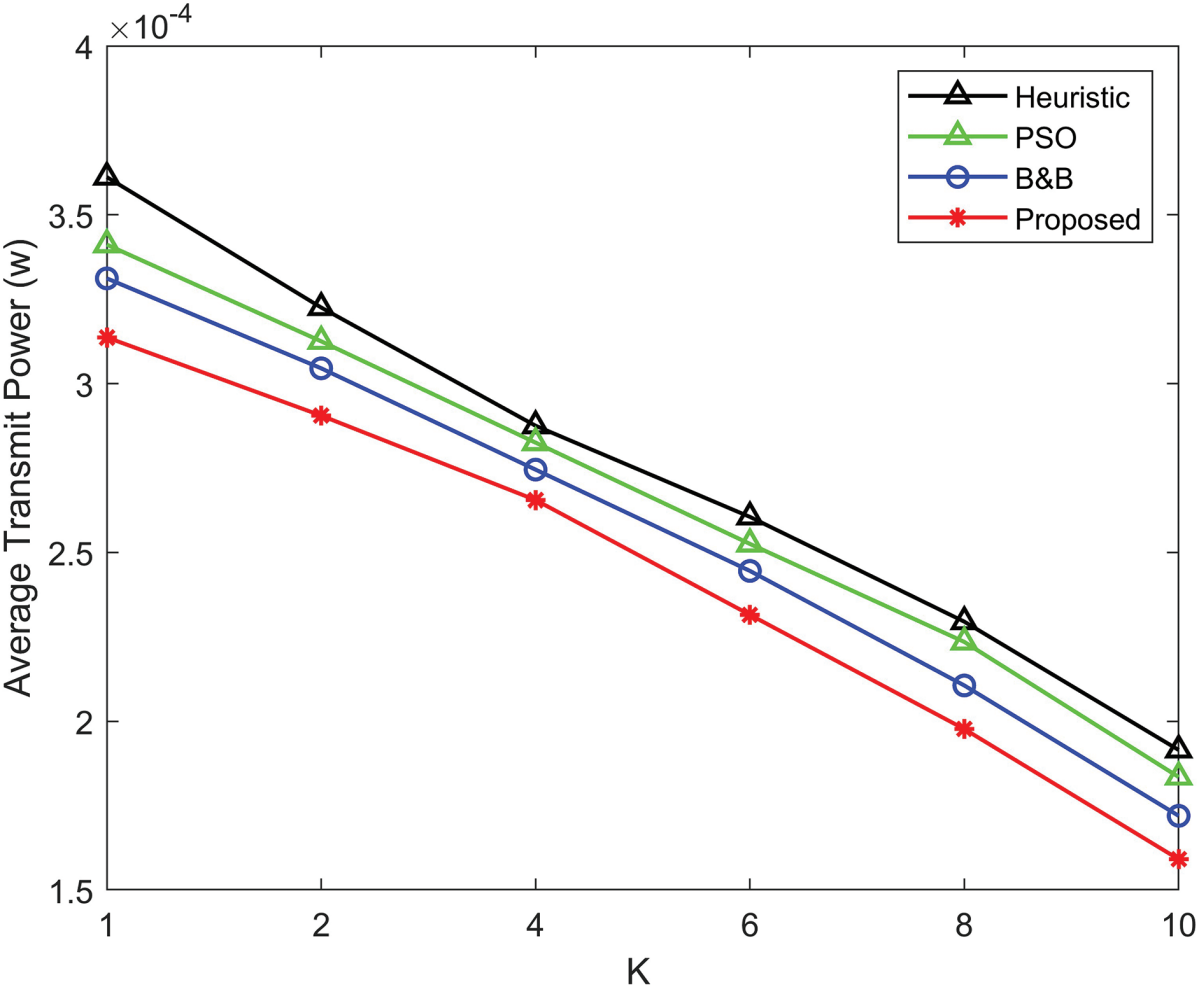
Comparison of average transmit power as a function of the Rician *K*-factor for the proposed power allocation algorithm, B&B algorithm, PSO algorithm, and heuristic algorithm.

Figure 7 illustrates the comparison of average transmit power between the proposed power allocation algorithm, the B&B algorithm, the PSO algorithm, and the heuristic algorithm as a function of the Rician *K*-factor. The Rician *K* factor characterizes the ratio between the LoS signal and the scattered signals in Rician fading environments. As the *K* factor increases, indicating stronger LoS conditions the average transmit power decreases. However, the power allocation algorithm consistently outperforms the B&B, PSO and heuristic algorithms, requiring less transmit power across all *K* values. This shows the efficiency of the power allocation algorithm, particularly at higher *K* factors, where the performance gap between the proposed power allocation algorithm and the B&B, heuristic and PSO algorithms indicates a more power-efficient solution in environments with strong LoS components.

**Figure 8:**
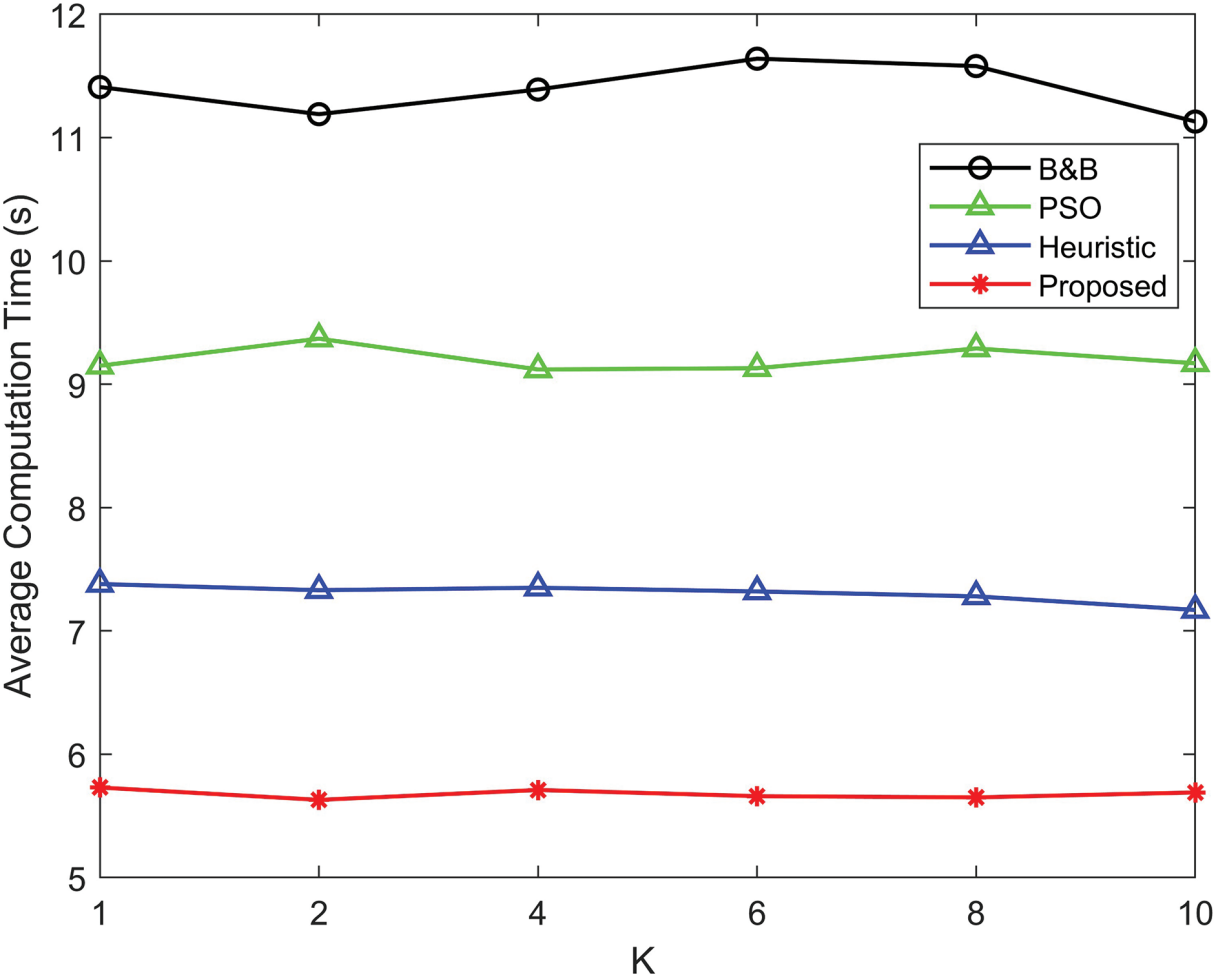
Comparison of average computation time as a function of the Rician *K*-factor for the proposed power allocation algorithm, B&B algorithm, PSO algorithm and heuristic algorithm.

Figure 8 compares the average computation time for different power allocation algorithms as a function of parameter *K*. The proposed algorithm achieves the lowest average computation time across all values of *K*, highlighting its computational efficiency compared to other methods. B&B exhibits the highest computation time, followed by PSO and heuristic algorithms, respectively, indicating their relative computational inefficiencies.

**Figure 9:**
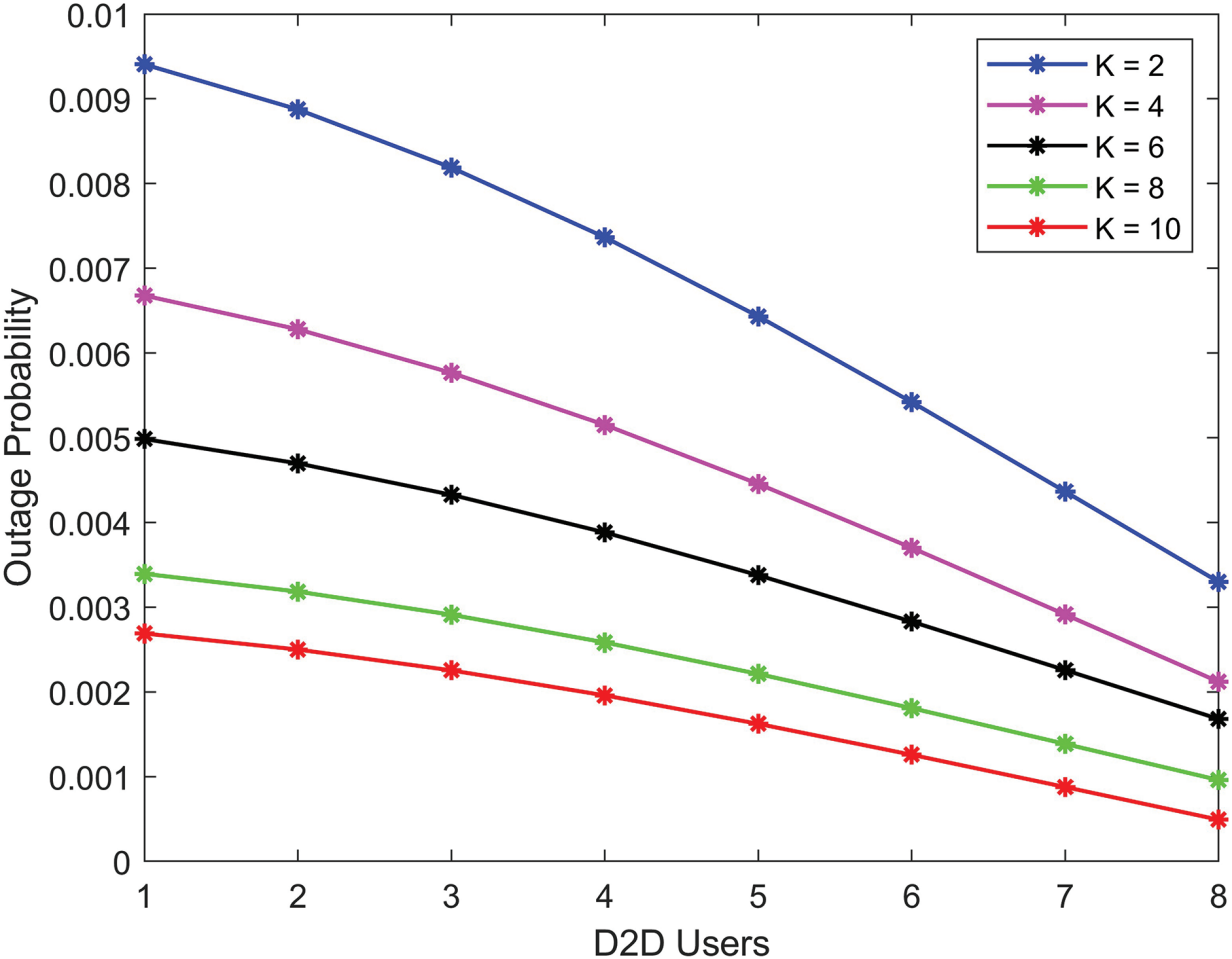
Outage probability for D2D communication (lower outage probability with higher *K*-factors as the number of users increases).

The Fig. 9, demonstrates that as the Rician *K*-factor increases, the outage probability of D2D communication improves. Because of the robust LoS communication and reduced multi-path propagation loss. We see that the quality of communication greatly increases due to the LoS between D2D communications, resulting in increased data rates for users.

**Figure 10:**
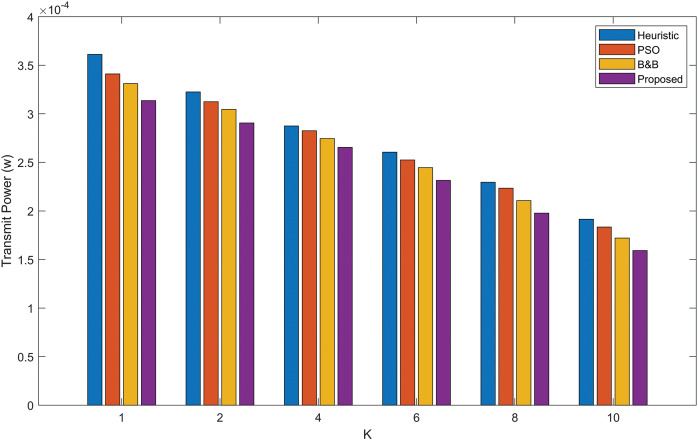
Optimal average D2D transmit power under URLLC with various Rician *K*-factors.

Figure 10 compares the average transmit power performance of the proposed power allocation algorithm, B&B, PSO, and heuristic algorithms across different values of *K* under URLLC requirements. The power allocation algorithm consistently demonstrates lower transmit power consumption compared to the other algorithms, highlighting its efficiency in optimizing power allocation in D2D communication scenarios with URLLC constraints. The power allocation algorithm performs better than the B&B, PSO and heuristic algorithms in terms of overall performance. Our algorithm minimizes power consumption and maintains low computational complexity for D2D communication in a URLLC scenario under the Rician fading, demonstrating the effectiveness of our approach.

## Conclusion

In this paper, we have proposed an algorithm based on derivative to optimize the power allocation in D2D communication. This research focused on analyzing the uplink D2D communication in a single-cell environment within the cellular system, with a particular emphasis on investigating the characteristics of the Rician fading. We defined an optimization problem related to power allocation, with the objective of maximizing the minimal achievable rate in D2D communication. To solve the non-convex optimization problem, we introduced an algorithm based on a derivative that iteratively converges toward the optimal solution. Our proposed power allocation algorithm aims to optimize power allocation in D2D communication while considering the URLLC with Rician fading. The power allocation algorithm iteratively reaches an optimal solution and is then subjected to a comparison with the B&B, heuristic, and PSO algorithms. The power allocation algorithm exhibits notably lower computational complexity compared to the B&B algorithm, heuristic algorithm, and PSO algorithm. Future research can explore improving power allocation using advanced machine learning techniques, such as reinforcement learning, for more adaptive solutions. This study focuses on a single-cell environment, and future research will examine the algorithm in multi-cell environments with various fading models. Extending it to 6G and IoT systems for resource block allocation, and interference management, would address the growing demands for low latency and high reliability in future wireless networks.

## Appendix A

From Eq. (7)

(34) $$P_m^{OP} = \left\{ {\gamma _m^d \le {\gamma _0}} \right\} = \Pr \left( {{{P_m^d\;S_m^d\;G_m^d} \over {\mathop \sum \nolimits_{n \in L} {p_{n,m}}P_n^c\;S_{n,m}^{cd}G_{n,m}^{cd}}} \le \;{\gamma _0}} \right)$$

(35) $$P_m^{OP} = 1 - \int_{{\gamma _{th}}}^\infty \left( {\mathop \int \limits_0^{{{{\gamma _d}} \over {{\gamma _0}}}} {f_{{\gamma _I}}}\left( {{\gamma _I}} \right)d{\gamma _I}} \right){f_{{\gamma _D}}}\left( {{\gamma _d}} \right)d{\gamma _d}$$

Eqs. (9) and (10) in Eq. (35) can be used to rewrite the D2D outage probability

(36) $$\eqalign{ {P_m^{OP} = 1 - \int\limits_{{\gamma _{th}}}^\infty \left( {\mathop \int \limits_0^{{{{\gamma _d}} \over {{\gamma _0}}}} {{{\gamma _I}^{{L_I} - 1}} \over {\bar \gamma _I^{{L_I}}\left( {{L_I} - 1} \right)!}}exp\left( { - {{{\gamma _I}} \over {{{\bar \gamma }_I}}}} \right)d{\gamma _I}} \right)} \hfill \cr {\left( {{{\left( {K + 1} \right)} \over {{{\bar \gamma }_d}}}\;{e^{\left[ { - K - {{\left( {K + 1} \right){\gamma _d}} \over {{{\bar \gamma }_d}}}} \right]}}{I_0}\left( {2\sqrt {{{K\left( {K + 1} \right){\gamma _d}} \over {{{\bar \gamma }_d}}}} } \right)} \right)} \hfill \cr }$$

Solving Eq. (36) inner integral and obtaining the outage probability as

(37) $$\eqalign{& {P_m^{OP} = 1 - {Q_1}\left( {\sqrt {2K} ,\sqrt {{{2\left( {K + 1} \right){\gamma _{th}}} \over {{{\bar \gamma }_d}}}} } \right) + {{K + 1} \over {{{\bar \gamma }_d}}}\sum\limits_{j = 0}^{{L_I} - 1} {1 \over {j!}}\int\limits_{{\gamma _{th}}}^\infty {{\left( {{{{\gamma _d}} \over {{\gamma _0}{{\bar \gamma }_I}}}} \right)}^j}} \hfill \cr &{exp\left[ { - K - \left( {{1 \over {{\gamma _0}{{\bar \gamma }_I}}} + {{K + 1} \over {{{\bar \gamma }_d}}}} \right){\gamma _d}} \right]{I_0}\;\left( {2\sqrt {{{K\left( {K + 1} \right){\gamma _d}} \over {{{\bar \gamma }_d}}}} } \right)d{\gamma _d}} \hfill \cr }$$

${Q_1}\left( { \cdot , \cdot } \right)$ denotes the first-order Marcum *Q*-function. The outage probability can be expressed by solving the integral and using the *Q*-function to simplify the Eq. (37) (Nuttall, 1975).

(38) $$P_m^{OP} = 1 - {Q_1}\left( {\sqrt {2K} ,\sqrt {{{2\left( {K + 1} \right){\gamma _{th}}} \over {{{\bar \gamma }_d}}}} } \right) + {{{a^2}} \over {2K}}\sum\limits_{j = 0}^{{L_I} - 1} {{{e_j}} \over {j!}}{Q_{2j + 1,0}}\left( {a,q} \right)$$

where ${e_j} = {{exp\left[ { - K + {K \over {\left( {1 + {{{{\bar \gamma }_d}} \over {\left( {K + 1} \right){\gamma _0}{{\bar \gamma }_I}}}} \right)}}} \right]} \over {{{\left( {2 + {{2\left( {K + 1} \right){\gamma _0}{{\bar \gamma }_I}} \over {{{\bar \gamma }_d}}}} \right)}^j}}}$, $a = \sqrt {{{2K} \over {\left( {1 + {{{{\bar \gamma }_d}} \over {\left( {K + 1} \right){\gamma _0}{{\bar \gamma }_I}}}} \right)}}}$ and $q = \sqrt {2\left( {1 + K + {{{{\bar \gamma }_d}} \over {{\gamma _0}{{\bar \gamma }_I}}}} \right){{{\gamma _0}} \over {{{\bar \gamma }_d}}}}$. Where ${L_I} = 1$ (Yang & Alouini, 2002; Yao & Sheikh, 1990), the Eq. (38) can be expressed as

(39) $$P_m^{OP} = 1 - {Q_1}\left( {\sqrt {2K} ,\sqrt {{{2\left( {K + 1} \right){\gamma _{th}}} \over {{{\bar \gamma }_d}}}} } \right) + {{{a^2}} \over {2K}}exp\left[ { - K + {{{a^2}} \over 2}} \right]{Q_1}\left( {a,q} \right)$$

The interference-limited $\;{\gamma _{th}} = 0$, the Eq. (39) can be

(40) $$P_m^{OP} = {{exp\left[ { - K + {K \over {\left( {1 + {{{{\bar \gamma }_d}} \over {\left( {K + 1} \right){{\bar \gamma }_d}}}} \right)}}} \right]} \over {\left( {1 + {{{{\bar \gamma }_d}} \over {\left( {K + 1} \right){\gamma _0}{{\bar \gamma }_I}}}} \right)}}$$

## Appendix B

The derivation of $P_m^{d*}$ is as follows

(41) $${R_m} = \;{{{B_m}} \over {ln2}}\;\left[ {{C_m} - \sqrt {{1 \over {{T_m}{B_m}}}} \;f{Q^{ - 1}}\left( {\epsilon _m^*} \right)} \right]$$

(42) $${R_m}\;{\triangleq}{f_m}\left( {P_m^{d*}} \right) = {{{B_m}} \over {ln2}}\;\left[ {{C_m} - \sqrt {{1 \over {{T_m}{B_m}}}} \;f{Q^{ - 1}}\left( {\epsilon _m^*} \right)} \right]$$

(43) $${{d{f_m}\left( {P_m^{d*}} \right)} \over {dP_m^{d*}}} = {d \over {dP_m^{d*}}}\left( {{{{B_m}} \over {ln2}}\;\left[ {{C_m} - \sqrt {{1 \over {{T_m}{B_m}}}} \;f{Q^{ - 1}}\left( {\epsilon _m^*} \right)} \right]} \right)$$

where ${C_m} = \;\log \;\left( {1 + \;\gamma _m^d\;} \right)$

(44) $$\matrix{ {{d{f_m}\left( {P_m^{d*}} \right)} \over {dP_m^{d*}}} =  {{d \over {dP_m^{d*}}}\left( {{{{B_m}} \over {ln2}}\;\left[ {\log \;\left( {1 + \;{{P_m^{d*}S_m^d\;G_m^d} \over {\mathop \sum \nolimits_{n \in {\scr L}} {p_{n,m}}P_n^cS_{n,m}^{cd}G_{n,m}^{cd} + {\sigma ^2}}}\;} \right) - \sqrt {{1 \over {{T_m}{B_m}}}} \;f{Q^{ - 1}}\left( {\epsilon _m^*} \right)} \right]} \right)} \cr }$$

(45) $$\matrix{ {{d{f_m}\left( {P_m^{d*}} \right)} \over {dP_m^{d*}}} =  {{{{B_m}} \over {ln2}}\left( {{d \over {dP_m^{d*}}}\left[ {\log \left( {1 + {{P_m^{d*}S_m^dG_m^d} \over {\mathop \sum \nolimits_{n \in {\scr L}} {p_{n,m}}P_n^cS_{n,m}^{cd}G_{n,m}^{cd} + {\sigma ^2}}}} \right)} \right] + {d \over {dP_m^{d*}}}\left[ { - \sqrt {{1 \over {{T_m}{B_m}}}} f{Q^{ - 1}}\left( {\epsilon _m^*} \right)} \right]} \right)} \cr }$$

(46) $$\matrix{ {{d{f_m}\left( {P_m^{d*}} \right)} \over {dP_m^{d*}}} =  {{{{B_m}} \over {ln2}}\left( {\left[ {{1 \over {1 + {{P_m^{d*}S_m^d\;G_m^d} \over {\mathop \sum \nolimits_{n \in {\scr L}} {p_{n,m}}P_n^cS_{n,m}^{cd}G_{n,m}^{cd} + {\sigma ^2}}}}}} \right]{d \over {dP_m^{d*}}}\left[ {1 + \;{{P_m^{d*}S_m^d\;G_m^d} \over {\mathop \sum \nolimits_{n \in {\scr L}} {p_{n,m}}P_n^cS_{n,m}^{cd}G_{n,m}^{cd} + {\sigma ^2}}}} \right] + 0} \right)} \cr }$$

(47) $${{d{f_m}\left( {P_m^{d*}} \right)} \over {dP_m^{d*}}} = {{{B_m}} \over {ln2}}\left( {{{{{S_m^d\;G_m^d} \over {\mathop \sum \nolimits_{n \in {\scr L}} {p_{n,m}}P_n^cS_{n,m}^{cd}G_{n,m}^{cd} + {\sigma ^2}}}} \over {1 + {{P_m^{d*}S_m^d\;G_m^d} \over {\mathop \sum \nolimits_{n \in {\scr L}} {p_{n,m}}P_n^cS_{n,m}^{cd}G_{n,m}^{cd} + {\sigma ^2}}}}}} \right)$$

where $P_m^{OP*} = \left\{ {w_m^d \le {w_0}} \right\} = {{{w_d}} \over {{w_I}}} \le {w_0} = \Pr \left( {{{S_m^d\;G_m^d} \over {\mathop \sum \nolimits_{n \in {\scr L}} {p_{n,m}}P_n^cS_{n,m}^{cd}G_{n,m}^{cd} + {\sigma ^2}}} \le \;{w_0}} \right)$, Where ${w_d} = S_m^dG_m^d$ and ${w_I} = \mathop \sum \nolimits_{n \in {\scr L}} {p_{n,m}}P_n^c\;S_{n,m}^{cd}G_{n,m}^{cd}$. Where $P_m^{OP*}$ and ${{P_m^{d*}S_m^d\;G_m^d} \over {\mathop \sum \nolimits_{n \in {\scr L}} {p_{n,m}}P_n^cS_{n,m}^{cd}G_{n,m}^{cd} + {\sigma ^2}}}$ solve by outage probability Eq. (7).

(48) $${{d{f_m}\left( {P_m^{d*}} \right)} \over {dP_m^{d*}}} = {{{B_m}} \over {ln2}}\left( {{{P_m^{OP*}} \over {1 + \;P_m^{OP}}}} \right).$$

## Supplemental Information

10.7717/peerj-cs.2712/supp-1 Supplemental Information 1 D2D Matlab Code.

## Additional Information and Declarations

### Data Availability

The following information was supplied regarding data availability:

The code is available in the Supplemental File.
